# The Virtual Teacher (VT) Paradigm: Learning New Patterns of Interpersonal Coordination Using the Human Dynamic Clamp

**DOI:** 10.1371/journal.pone.0142029

**Published:** 2015-11-16

**Authors:** Viviane Kostrubiec, Guillaume Dumas, Pier-Giorgio Zanone, J. A. Scott Kelso

**Affiliations:** 1 EA-4561 PRISSMH, Université de Toulouse, UPS, Toulouse, France; 2 Center for Complex Systems and Brain Sciences, Florida Atlantic University, Boca Raton, FL, United States of America; 3 Intelligent Systems Research Centre, University of Ulster, Derry ~ Londonderry, N. Ireland; Universiteit Gent, BELGIUM

## Abstract

The Virtual Teacher paradigm, a version of the Human Dynamic Clamp (HDC), is introduced into studies of learning patterns of inter-personal coordination. Combining mathematical modeling and experimentation, we investigate how the HDC may be used as a Virtual Teacher (VT) to help humans co-produce and internalize new inter-personal coordination pattern(s). Human learners produced rhythmic finger movements whilst observing a computer-driven avatar, animated by dynamic equations stemming from the well-established Haken-Kelso-Bunz (1985) and Schöner-Kelso (1988) models of coordination. We demonstrate that the VT is successful in shifting the pattern co-produced by the VT-human system toward any value (Experiment 1) and that the VT can help humans learn unstable relative phasing patterns (Experiment 2). Using transfer entropy, we find that information flow from one partner to the other increases when VT-human coordination loses stability. This suggests that variable joint performance may actually facilitate interaction, and in the long run learning. VT appears to be a promising tool for exploring basic learning processes involved in social interaction, unraveling the dynamics of information flow between interacting partners, and providing possible rehabilitation opportunities.

## Introduction

**Fig 1:**
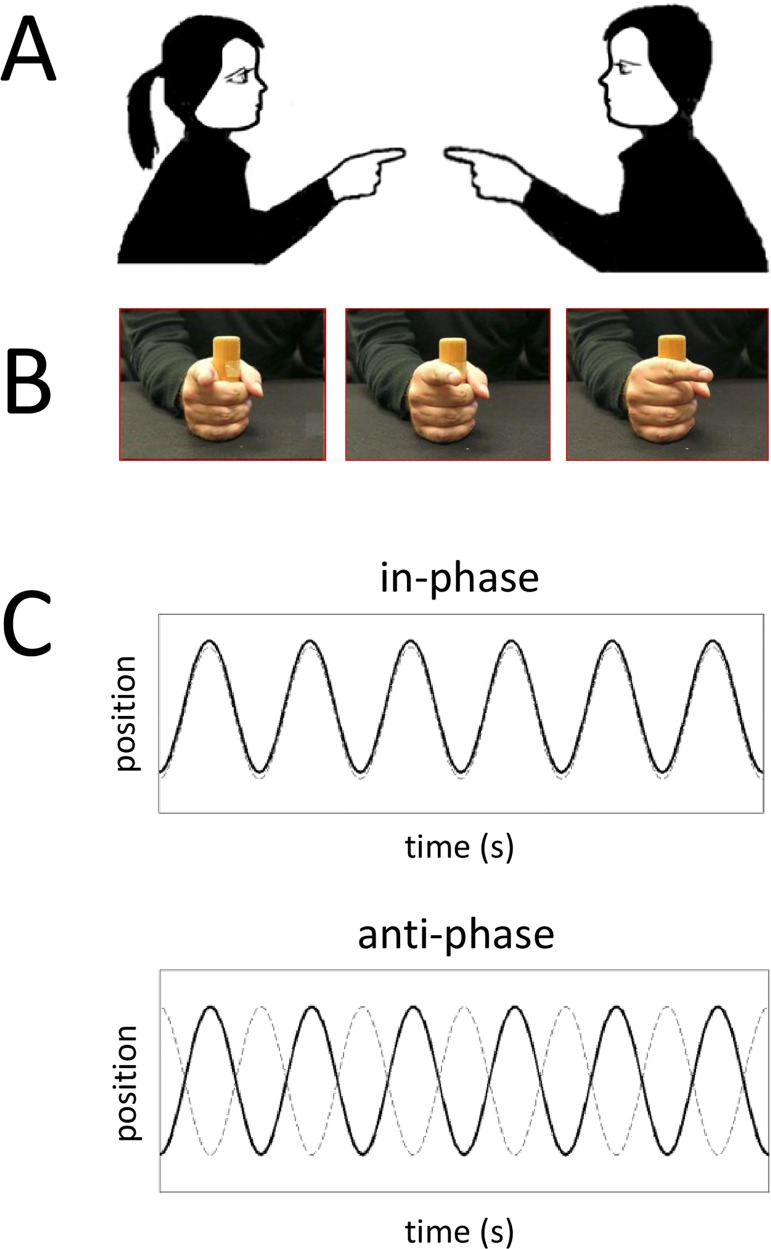
Coordination Paradigm. Typical inter-personal coordination paradigm (A) where visual information about finger movement is exchanged between the two partners (B), and the resulting finger movement is recorded as a function of time (C), here depicting in-phase and anti-phase coordination between participants. When in-phase, fingers move in the same direction, whereas when anti-phase, they move in opposite directions.

In many real life situations, new behaviors are generated for the first time with the assistance of a more expert partner [1–2]. A good example is the generation of new patterns of inter-personal coordination during joint action, such as in playing collective sports or in performing ballroom dancing. In the present contribution we exploit a social situation that typically involves two participants [3–8]. In one variant [3–4], the partners watch each other whilst simultaneously producing rhythmic motions of the index finger in the horizontal plane (Fig 1A and 1B). As a consequence of the visually mediated inter-personal coupling (Fig 1A), two spontaneously stable patterns of coordination are observed: the more stable in-phase and the less stable anti-phase (Fig 1C).

Operationally, coordination patterns may be captured by the relative phase (RP), which assesses the temporal gap between the oscillatory displacements of two limbs [9–10]. Measured in degrees, RP amounts to 0° for in-phase and of 180° for anti-phase. A pattern is said to be stable if it recovers its value after a perturbation. The more stable a pattern is, the less RP varies [10]. Experiments (e.g. [9,11]) and theory (e.g., [10–13]) show that the less stable anti-phase pattern tends to switch to in-phase as a result of loss of stability.

In the present work, one partner is a naive learner and the other plays the role of a teacher. Importantly, the goal of the teacher is to co-produce with the learner a coordination pattern that is distinct from in-phase and anti-phase. We manipulate how strongly the teacher adjusts to the learner and quantify, using information-theoretic measures, how information mediating inter-personal coupling flows from one partner to another. The learner’s task is to first identify and then learn the pattern the teacher wants him/her to learn in the absence of any explicit, verbal communication about the intended inter-personal coordination pattern.

**Fig 2:**
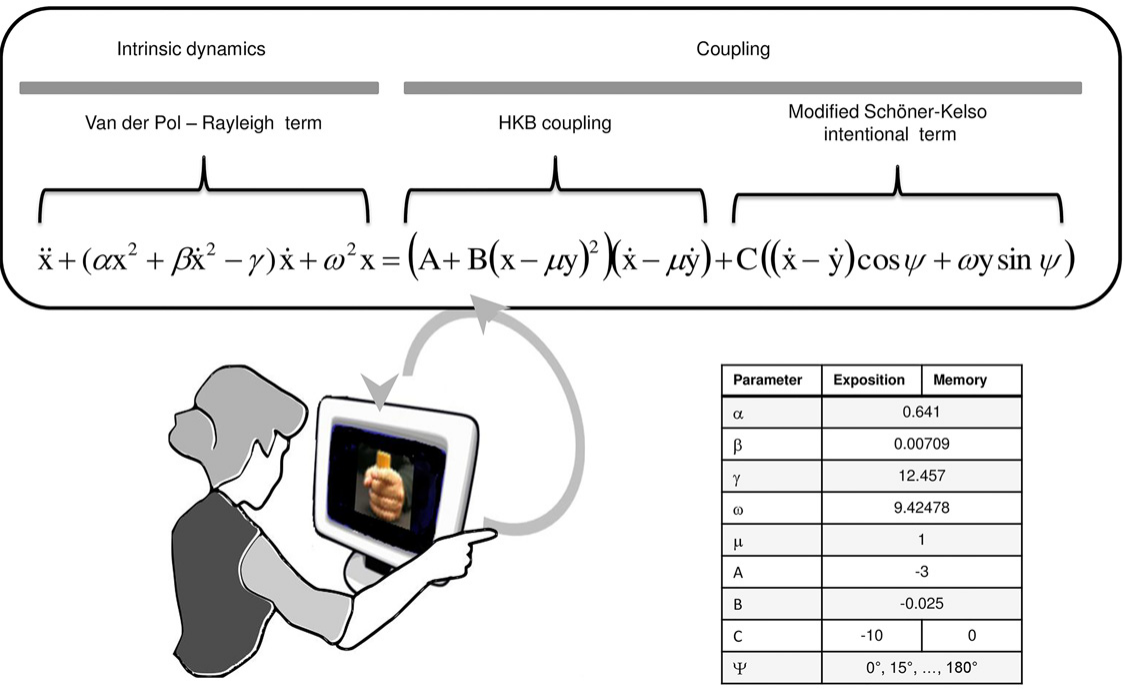
Virtual Teacher setup. The current finger position of the human participant is sent to a model governing the current finger position of the VT. In an Exposition period of the experiment, the VT is programmed to coordinate with the participant so that a relative phase specified by the VT (Ψ) is eventually co-produced. In a Memory period, the modified Schöner-Kelso intentional term of the VT is set to 0°, whereas the HKB coupling term is set either to nonzero (Experiment 1) or to zero (Experiment 2). See text for details.

There is one twist to the present research. In order to reliably manipulate the interaction between teacher and learner, a real human teacher is replaced by a model-driven avatar. The avatar—an animated image displayed on a computer screen (Fig 2)—is controlled by an empirically verified theoretical model of coordination dynamics which reciprocally couples with a real-life partner’s finger movements (Fig 1A). Given that the design of the model-driven avatar is known in its entirety by manipulating its parameters we can study their effects on emergent patterns of coordination in the human-model system. Elsewhere we refer to this situation as Virtual Partner Interaction [14] or the Human Dynamic Clamp [15–16]. The original Human Dynamic Clamp [14] used the well-known Haken-Kelso-Bunz (HKB) model of coordination dynamics [13]. Here we realize the model-driven avatar by extending the Schöner-Kelso [17] model of coordination learning [15–16]. In the present context of learning, we call the model-driven avatar a Virtual Teacher (VT).

What is known to date about the dynamics of learning coordination patterns comes from twenty-five years of studying individual mechanisms—a research program begun by Zanone and Kelso [18–20] and developed by many others [21–29]. When an individual is asked to oscillate both index fingers in the horizontal plane, spontaneously stable in-phase and anti-phase inter-limb coordination patterns are observed [9]. These stable states emerge before any learning occurs and preclude all others patterns from being readily produced [18–22][23][30–31] and accurately perceived [32]. The stable patterns may be said to exert constraints on coordination, and the learning of new RPs may arise only by overcoming such constraints through practice [18–20][25][33]. Before learning begins, it is therefore important to identify and quantify pre-existing stable patterns using the so-called scanning procedure [18–20][23][31].

In the classical scanning procedure, an individual is exposed to all possible RPs, ranging from 0° to 180° by steps of 15° and asked to produce the required pattern by coordinating finger or limb movements in such a way that the time lag between the oscillatory limb movements achieves a specified value. Each RP is visually displayed using an appropriate delay between the onsets of two light emitting diodes (LED), one for each finger. Individual subjects are asked to reproduce each displayed RP by synchronizing movement with the LED onset. It is important to note that no performance feedback is provided in the scanning procedure: Scanning does not aim to address learning, *per se*, but rather aims to capture the pre-existing behavioral repertoire (so-called “intrinsic dynamics”, see [11], [18], [34]) of each individual participant before learning begins.

An advantage of scanning is that it allows one to identify patterns that individuals are unable to perform initially and to select one of them as the pattern to be learned (e.g., 90° RP). In the Zanone-Kelso procedure, practice is administered only after scanning. At practice, the learner attempts to produce the to-be-learned RP; Knowledge of results is then provided about the accuracy and stability of the performed pattern. Experimental findings show that stable RPs identified through scanning before learning affect (and are affected by) the learning of new patterns [18–20][25][35]. Moreover, new pattern learning appears to be triggered by the internalization of required movement-related feedback rather than by the generation of required motor commands [36–37].

Returning now to inter-individual learning, the present experiments may be viewed as a proof of concept that the VT-human system is able to perform coordination patterns that two interacting naive humans would have difficulty to coproduce and learn by themselves. We exploit the Zanone-Kelso scanning procedure used for individual learning, but now in an inter-personal learning situation, where the VT replaces a human “teacher”. In the first experiment, scanning is administered in order to capture how spontaneously stable 0° and 180° RPs (hypothesized to belong to the learner's behavioral repertoire) interplay with RPs specified by the VT. On the basis of scanning results, a to-be-learned pattern is selected and used in the second learning experiment. In both experiments, we manipulate how the VT adjusts itself to the learner and test whether such changes are related to the amount of information exchanged between VT and human.

The experimental set-up is shown in Fig 2. The HKB equations simulate the motion of two effectors, *x* and *y*, as two, nonlinearly coupled nonlinear oscillators [13]. Constructing the original version of the HDC, one of the simulated oscillators (*y* in Fig 2) was replaced by a real human limb [14], whereas the other simulated oscillator (*x* in Fig 2) played the role of a virtual partner. In Fig 2, the left side of the virtual teacher equation (composed of Van-der-Pol and Rayleigh terms established by empirical studies of rhythmic movements [38–40]) sets the avatar in motion. The variables, $x,\;\dot{x}$, and $\ddot{x}$, refer to virtual limb position, velocity and acceleration, respectively, the constants *α*, β, γ and ω to their parameters. The right part of the equation implements the coupling coming from the human to the VT, where *y* pertains to human position, $\dot{y}$ to velocity, A, B and μ to parameters (Fig 2, right).

The right side of the VT equation is composed of two coupling expressions: the HKB coupling term and a modified Schöner-Kelso intentional term [15]. The HKB term gives rise to two stable solutions at 0° and 180° RP, the parameter μ serving to scale the response of the human’s movements to the dynamic range of the virtual teacher’s. The modified Schöner-Kelso intentional term [17] produces a shift of the co-produced pattern toward any arbitrary RP, specified as Ψ inside the VT equation. We may refer to the RP specified by the VT as the pattern to be learned by the human partner. The coupling strength between human and VT is manipulated through the parameter C: The higher the absolute value of C is, the stronger the shift performed by VT toward the Ψ value. When both the Schöner-Kelso term and the HKB term are cancelled, by setting the corresponding parameters to zero (C = 0, or A = B = 0, respectively), VT acts independently from the human, basically as a virtual metronome.

During the VT-human interaction, both the motion produced by the VT (viz. left side of equation) and the coupling coming from the human to the VT (viz. right side of equation) can be manipulated. In the present experiment, the Van der Pol-Rayleigh parameters are constant (though they can be made time-dependent, cf.[15]). The present experimental design bears only on the manipulation of the coupling parameters.

Both experiments employ the same basic procedure. An RP (Ψ) is inserted into the modified Schöner-Kelso term and the participant is asked to move the index finger whilst watching the avatar. In each trial, an Exposition-Memory procedure, akin to the synchronization-continuation paradigm [41–44] is used. In the Exposition period, C is set at a non-zero value and the learner's task is to produce the to-be-learned inter-personal coordination pattern. In the Memory period, C is lowered to zero and the learner is encouraged to continue and maintain the just-produced RP.

The first, scanning experiment aims to test whether the VT can be used to dominate inter-personal coordination during the Exposition phase by attracting the co-produced RP toward any desired value, Ψ. We quantify the influence of spontaneously stable patterns (0° and 180°) on the performance of the VT-human system when all possible RPs, ranging from 0° to 180° in steps of 15°, are fed into the VT equation. Then, during the Memory phase of each trial, the modified Schöner-Kelso term is set to zero (C = 0) whereas the HKB term is left intact at a non-zero value (A≠0 and B≠0), thus attracting the VT-human system toward 180° or 0° RP. The VT-human system, just-driven away from their spontaneously stable patterns by the Schöner-Kelso term, is expected to converge toward these stable states. In the present contribution, we show that at Exposition VT can attract co-produced RPs toward any value, and that information flow rises when the VT-human system converges toward 0° or 180° RP during the early part of the Memory phase.

In the second experiment, we test whether the VT can be efficiently used as a ‘teacher’ to specify a new pattern for the learner to identify, co-produce, and eventually learn. An RP (Ψ = 90°) selected on the basis of the scanning results of Experiment 1 is used as the to-be-learned pattern in Experiment 2. For learning to occur, the learner has to engage in the inter-personal pattern specified by the VT at Exposition despite the influence that his/her own preferred tendency to 0° and 180° may exert on the co-produced outcome. If the VT learning technique is to prove efficient, exposure to the to-be-learned RP should eventually be consolidated in the learner's memory, allowing him or her to sustain the just-performed pattern on his/her own without assistance from the VT. We show that at Exposition the VT is able to attract the co-produced RP toward the to-be-learned value (90°). At Memory, when the learner is asked to coproduce the RP specified by the VT when the coupling is completely removed (A = 0, B = 0, C = 0), we show that information flow from the VT is initially enhanced and that the pattern learned persists.

In summary, the present work aims to identify conditions that enhance the amount of information exchange between a human learner and a VT, the design of which is based upon empirically verified models of coordination dynamics. The VT is used not only to specify novel patterns for the human to discover and learn but also to check whether our theoretical model of simple forms of perceptuo-motor learning stands up to interaction with a real human.

## Experiment 1

### Method

#### Participants

Six unpaid volunteers, 4 males and 2 females, aged between 20 and 44 years took part in the study. All were self-proclaimed right-handers, naive to the purpose of the study. Participants reported normal or corrected-to normal visual acuity and had no physical or neurological impairments. Participants provided written informed consent prior to the research. The study was approved by the Internal Review Board at Florida Atlantic University and conformed to the principles expressed in the Declaration of Helsinki.

#### Material and apparatus

Participants were seated in a dark room, with the ulnar side of the right forearm resting against a U-shaped support (21.5 × 8 × 4 cm) positioned parallel to a table. Participants grasped a vertical wooden cylinder (4.5 × 3 cm) with their right hand, leaving only the right index finger in extension. The distal point of the index finger was inserted into the circular orifice (2 cm diameter) of another wooden block. The latter was connected through two metallic bars to a vertical, freely rotating metallic stick (18 cm length) whose angular displacement was captured by a linear potentiometer. The entire arrangement constituted a manipulandum, which was fixed on top of a plexiglas box (30.5 × 31.5 × 20 cm), positioned to the right of a screen, about 50 cm away from the midline of the participant. The manipulandum restricted the movement of the index finger to the horizontal plane, allowing a full-range of friction-free flexion-extension motion about the metacarpo-phalangeal joint.

The output of the potentiometer was sampled at 1000 Hz using a National Instruments A/D converter. The signal was down-sampled offline to 500 Hz and used as a continuous input into a computer which contained the VT program implemented on C++ using cross-platform IDE Code Blocks. A simplified version of the Human Dynamic Clamp is available free on Git-Hub: https://github.com/crowd-coordination/web-vpi. The velocity of the human finger was numerically computed using a 3-point differentiation algorithm and, together with position data plugged into the VT equation. The differential equation returned instantaneous VT acceleration which was integrated using a 4^th^ order Runge-Kutta method at 500 Hz to provide VT velocity and position. A maximum delay of 2 ms occurred between data acquisition and computation of the model output.

To create the animation of VT finger movement, a high-speed camera recorded a human male producing flexion-extension finger motion in the horizontal plane. A complete cycle of movement provided 119 images (17 × 13 cm) indexed by their position. The instantaneous position of the VT was used to select one of the 119 position-indexed images, which was displayed in the center of the screen (59 cm diagonal). The screen animation was refreshed at 100 Hz during the experiment and looked just like an ordinary video display of a real finger in periodic motion. An auditory tone of 440 Hz and 0.1 s duration was used as a pacing signal.

For the entire experimental session, all VT parameters were fixed (see table in Fig 2), except for the value of C and the RP (Ψ) specified by the VT. At Exposition, C was set at a value strong enough to attract the co-produced RP toward the RP of the VT. Pilot studies revealed that the VT attracts the RP close to the required value when C is lower than -4; here it was set at -10. During Memory, C was set at 0, leaving only the HKB coupling parameters to attract the co-produced RP toward 0° or 180°. In each scanning trial, one of thirteen RPs, ranging from 0° to 180° by steps of 15°, was plugged into the VT equation (i.e.,Ψ). It was thus possible to assess the ability of the human learner to co-produce the RP specified by the VT at Exposition and to determine during the Memory period if the pattern persisted despite the attraction of VT toward 0°.

#### Procedure

Each trial was composed of three periods: Pacing, Exposition and Memory, each lasting 6s, 20s, and 11s, respectively. A fixation cross appeared at the center of the screen until the participant pressed the keyboard space bar to start a trial. During the pacing period an auditory tone provided the required movement frequency (1 Hz). Participants were instructed to produce peak flexion on each beat and then to maintain the frequency throughout the rest of the trial. As soon as the pacing signal was turned off, the Exposition phase began (C ≠ 0) and the moving VT finger appeared on the screen. Participants were instructed to produce one complete cycle of finger movement for each complete movement cycle of the VT’s finger movement. Then the Memory phase started (C = 0). Participants were told to maintain coordination even if they noticed a change in VT behavior (“keep doing what you did”). This instruction served to introduce a competition between the memory of the just-performed pattern and the tendency shared now by the VT-human system to co-produce 0° RP.

Four scannings were administered in a row, separated by a one-minute pause. Each scanning consisted of thirteen trials, each displaying a distinct value of RP specified by the VT (Ψ). The required relative phases were presented at random and ranged from 0° to 180° in steps of 15°. The duration of the entire experimental session was approx. 45 min.

#### Data reduction and analysis

Potentiometer signals representing finger displacement of the human and of the VT were mean-centered, detrended, low-pass filtered using a second order dual-pass Butterworth filter with a cutoff frequency of 20 Hz, and normalized, cycle by cycle, between -1 and 1 values, for Exposition and Memory separately. After this preprocessing, the continuous relative phase (RP) between human and VT movement was computed using a continuous Hilbert transform. To avoid transients, the first and last seconds of the time series were removed from the analysis, leaving 30 s of each trial for analysis (20s of Exposition and 10s of Memory). The RP was then separated in three periods, the 20 s of Exposition, the first 5 s of Memory (M1) and the last 5 s of Memory (M2). We expected that after setting C at zero, the RP would shift toward in-phase. Memory was thus divided into two separate periods, in order to distinguish the period of transient shift (M1) from the period of potential stabilization at 0° RP (M2).

For each of the three periods, mean RP and the corresponding circular variability were calculated, in order to assess the produced RP and its stability, respectively. Constant error (CE) was computed as the smallest distance between the produced and the RP specified by VT, corresponding either to the displayed RP or to its symmetry pattern in the interval zero to 360° (eg. 90 and–90°).

#### Transfer entropy

For each time series of position, transfer entropy (TE) was computed to quantify the information flow from VT to the learner and from learner to the VT. TE is an informational measure developed by Schreiber [45] to capture the amount and the direction of information flow exchanged between two systems, X and Y. TE measures how much of the future of a signal is explained by the past of the other signal but not by its own past. Basically, if X is the source and Y the target, TE assesses how much uncertainty about the next state of Y is reduced or how much predictability is gained by knowing X’s past activity, in addition to what is already known about Y’s past [46]. TE captures information flow from X to Y over time, not the information that is shared between these systems because of a common history and common input [47–48]. The formula of TE from X to Y may be represented as a difference between two conditional entropies [45–47] (see Eq 1):

$$T_{X\to Y}=H\left(Y_{i+\tau }|Y_{i}^{\left(k\right)}\right)-H\left(Y_{i+1}|Y_{i}^{\left(k\right)},X_{i}^{\left(m\right)}\right)$$ (1)

where *i* indicates a given instant, τ the time lag, *k* the Y past state’s vector and *m* the X past state’s vector (see appendix). Transfer entropy is null, that is X and Y are independent, if the next state of Y depends on *k* previous states of Y but not on the *m* previous states of X. Transfer entropy is positive if including the information about past states of X improves the prediction of the next state of Y beyond the prediction based on past states of Y. The improvement in prediction is calculated in bits. The formula of TE from Y to X is the same, except that Y is replaced by X and conversely.

To compute TE we used a technique introduced by [47] that employs the first minimum of the autocorrelation function as the delay of embedding and k = 5 as dimension. One criterion for the dimension is that in order to cover the attractor, it should be at least 2v+1, where v pertains to the dimensionality of the coordination system. The dimensionality, i.e. measure of the correlation dimension via the correlation integral, C (L), is provided in Kay, Saltzman and Kelso [39]. The motivation for the choice of k is that it should be greater than or equal to the dimension (v) of the dynamics (which are typically unknown—and were unknown until KSK), One criterion for k is that it should be at least 2v+1, in order to cover the attractor. The authors found that the correlation dimension was a bit over v = 1 similar values found for a limit cycle (i.e. a closed curve) plus a small noise. Therefore our choice of k = 5 veers on the conservative side.

With the help of several simulations, the τ parameter was chosen as the first zero crossing of the autocorrelation decay function [49]. Past state’s vectors were 5 values long.

#### Statistical analysis

To assess how the co-produced RP evolves as a function of RP specified by the VT (Ψ), a 3 (Period = {Exposition, Memory M1, Memory M2}) × 13 (Pattern = {0°, …, 180°}) ANOVAs on RP, CE, and circular variance were carried out with repeated measures on all factors. If necessary, this analysis was complemented with polynomial contrasts testing for data trends. To capture whether Ψ influenced informational flow, a 2 (Direction = {from VT to Human, from Human to VT} × 3 (Period) × 13 (Pattern) ANOVA on TE was carried out, followed, if required, by *t*-tests with Bonferroni correction for repeated comparisons. For all results, only significant effects at *p < 0*.05 are reported along with corresponding estimates of effect size (η^2^).

### Results

#### Visual inspection of individual data

**Fig 3:**
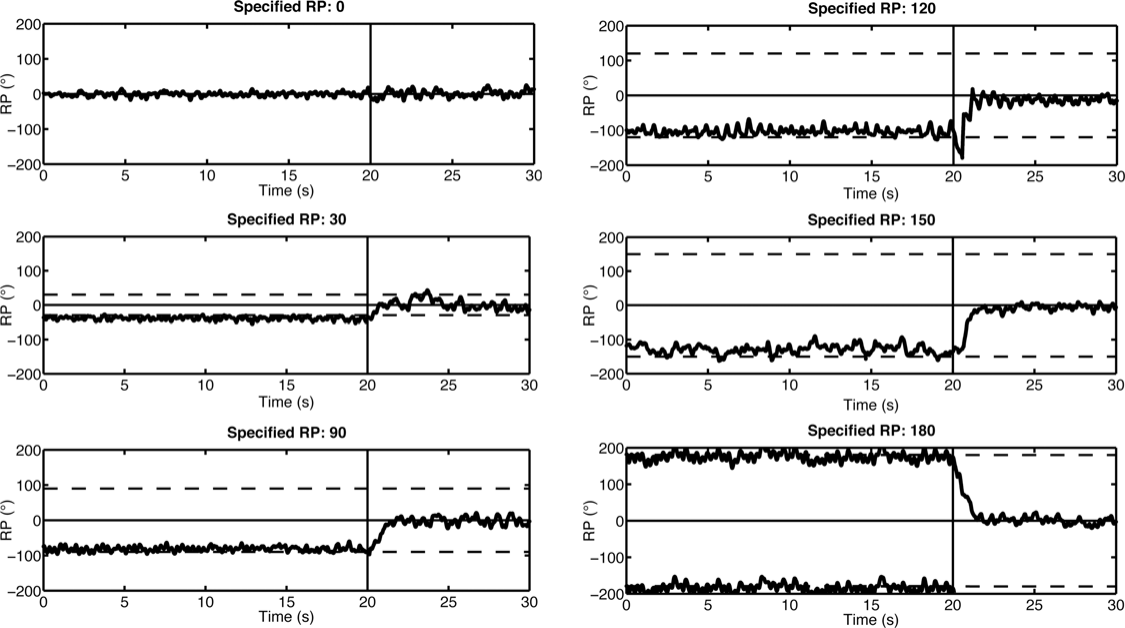
Individual data at scanning. Individual data showing the produced RP (in°) as a function of time in six scanning trials. Each panel pertains to a pattern specified by the VT (RP = {0, 30, 90, 120, 150, 180°}); dotted lines represent the specified RP. The vertical bar in each panel denotes the end of the Exposition and the beginning of the Memory period.

Visual inspection of individual data (see Fig 3) revealed that during Exposition, the produced RP remained close to the RP specified by the VT, suggesting that C was effective in producing the RP specified by the teacher. As soon as C switched to zero, at the beginning of the Memory period, the RP rapidly converged toward 0°.

#### Produced RP

**Fig 4:**
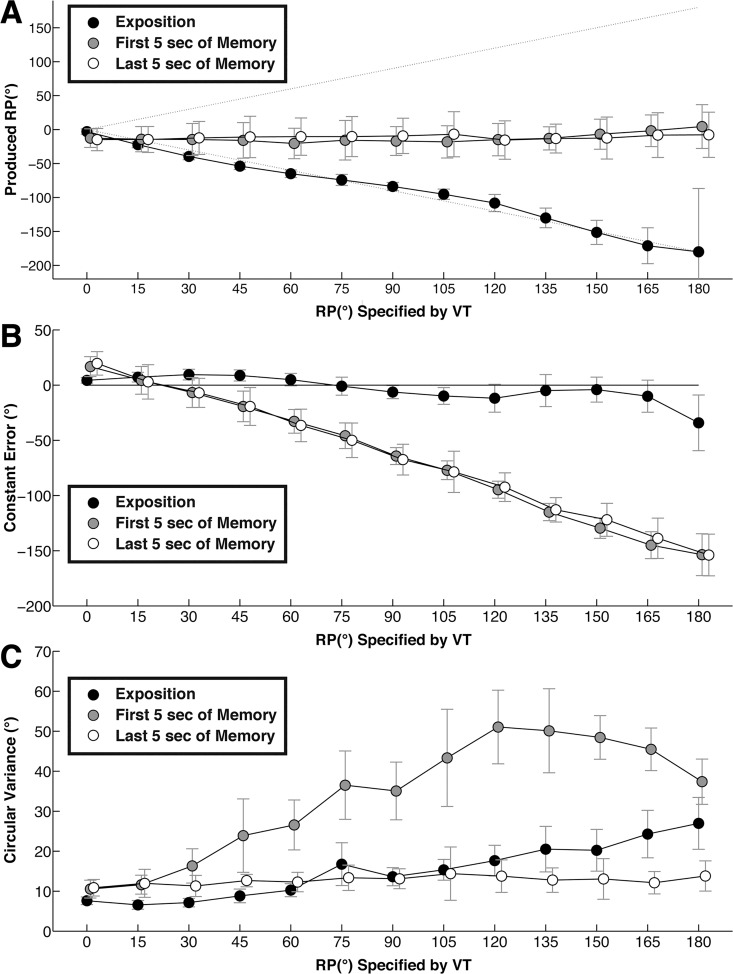
Scanning results. A: RP produced during the Exposition period (black circles), the first 5 s of the Memory period (gray circles) and the last 5 s of the Memory period (white circles), as a function of RP specified by the VT. B: Constant error produced during the Exposition period (black circles), the first 5 s (gray circles) and the last 5 s of the Memory period (white circles) as a function of specified RP. C: Circular variance produced during the Exposition period (black circles), the first 5 s (gray circles) and the last 5 s of the Memory period (white circles) as a function of specified RP. Vertical bars indicate between-subject standard error.

The produced RP is displayed as a function of RP specified by the VT in Fig 4 (top panel A). The ANOVA revealed a main effect of Period, *F*(2,10) = 260, p < 0.01, η^2^ = 0.98, of Pattern, *F*(12,60) = 17.20, *p <* 0.01, η^2^ = 0.79, and a significant Period × Pattern interaction, *F*(24,120) = 30.3, *p <* 0.01, η^2^ = 0.98. Whereas at Exposition, polynomial contrasts indicated that the produced RP decreased linearly as a function of the specified one, *F*(1,6) = 91.62, *p <* 0.01, η^2^ = 0.90, no effect of Pattern was present at Memory (*ns* for M1 and M2), all produced RPs remaining close to the 0° pattern. The produced RP at Exposition matched closely the specified pattern, illustrated by the dotted line in Fig 4 (panel A). At Memory RP converged toward a 0° value, irrespective of the specified pattern.

#### Constant error

CE is displayed in Fig 4 as a function of RP specified by VT (middle panel B). The ANOVA revealed a main effect of Period, *F*(2,10) = 246, p *< 0*.01, η^2^ = 0.84, of Pattern, *F*(12,60) = 625, *p <* 0.01, η^2^ = 0.38, and a significant Period × Pattern interaction, *F*(24,120) = 54.10, *p <* 0.01, η^2^ = 0.79. Polynomial contrasts indicated that in Exposition CE evolved as an M-shaped function, *F*(1,6) = 8.59, *p <* 0.03, η^2^ = 0.59 of 4^th^ order, whereas in Memory the error increased linearly as a function of Pattern, *F*(1,6) = 6.10, *p <* 0.05, η^2^ = 0.50; *F*(1,6) = 5.88, *p <* 0.05, η^2^ = 0.50 for M1 and M2, respectively. During Exposition, the curve of CE pictured in Fig 4 (panel B), took the form of an inverted S, located close to zero; at Memory it decreased linearly, reflecting the systematic production of 0° regardless of the previously performed pattern.

#### Circular variance

Circular variance is displayed as a function of the RP specified by the VT in Fig 4 (bottom panel C). The ANOVA revealed a main effect of Period, *F*(2,10) = 152, *p <* 0.01, η^2^ = 0.97, of Pattern, *F*(12,60) = 33.5, *p <* 0.01, η^2^ = 0.87, and a significant Period × Pattern interaction, *F*(24,120) = 11.70, *p <* 0.01, η^2^ = 0.701. Polynomial contrasts revealed that in Exposition circular variance increased linearly as a function of Pattern, *F*(1,5) = 13.06, *p <* 0.02, η^2^ = 0.72; in Memory M1 it followed a cubic trend, *F*(1,6) = 48.32, *p <* 0.01, η^2^ = 0.91.

#### Transfer entropy

**Fig 5:**
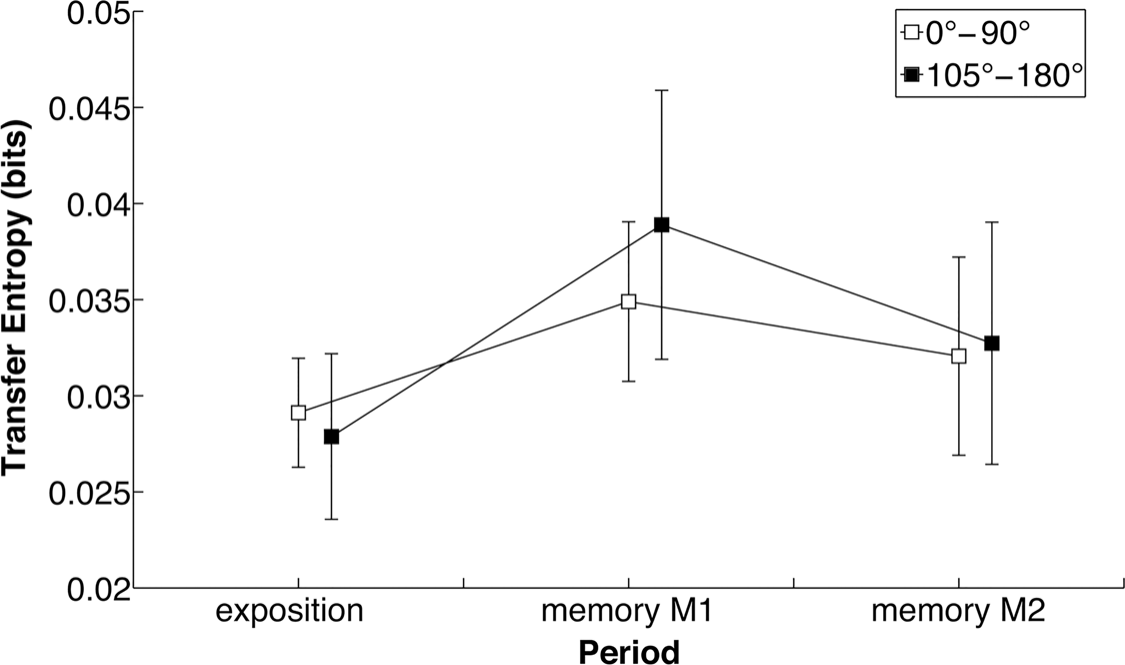
Transfer entropy at scanning. Transfer entropy as a function of Period (Exposition, Memory M1, Memory M2) for 0–90° patterns (white boxes) and 105–180° patterns (black boxes).

Transfer entropy is displayed as a function of RP specified by the VT in Fig 5. ANOVA revealed a main effect of Period, *F*(2,10) = 89.0, *p* < 0.01, η^2^ = 0.95, and a Period × Pattern interaction, *F*(24,120) = 2.63, *p* < 0.01, η^2^ = 0.35. There was neither a main effect nor an interaction effect for Direction. TE was larger at M1 than at Exposition (*p < 0*.01) and M2 (*p < 0*.01); and larger at M2 than at Exposition (*p < 0*.01). After grouping the patterns in two classes, corresponding to 0°-90° and 105°-180° patterns specified by the VT, a subsequent 3 (Period) × 2 (Class) ANOVA revealed a main effect of Period, *F*(2,10) = 33.3, *p* < 0.01, η^2^ = 0.87, and a significant Period × Class interaction, *F*(2,10) = 7.33, *p* < 0.01, η^2^ = 0.59. During M1, TE was larger for 105–180 than for the 0–90 patterns (*p < 0*.05). The informational flow, both from VT to human and from human to VT, increased when the produced pattern shifted from the RP specified by VT to 0° and variability increased. This effect was strongest for the class of 105°-180° patterns, that is, far from 0°.

### Conclusion and discussion

At Exposition, the VT was demonstrated to dominate inter-personal coordination: the produced patterns matched the RP specified by VT with high accuracy and stability, as shown by the curves of the produced RP and its variance. This finding establishes that VT can be used to stabilize the VT-human system at any RP. VT was able to interact with a real human, suggesting that it captures, computes and returns useful variables for a human in a relevant way. An artificial device—heart, kidney or synapse—may work within a living organism if two conditions are fulfilled: (1) the interaction between the corresponding natural component and the rest of the organism is underwritten by a specific law; and (2) this law is adequately simulated by the artificial device. In our case here, VT works in interaction with a human partner to the extent that inter-personal interaction is underwritten by lawful coordination dynamics, and the system is determined by a sufficient and necessary set of variables and parameters belonging to a relevant equation.

The scanning method provides insight into how, before any learning, the effect of RP patterns specified by the VT depends on spontaneously stable RPs present in the learner’s behavioral repertoire. At Exposition, the spontaneously stable RPs slightly affected the VT-human coordination as revealed by the small CE. In the range 0°-60°, produced RPs were higher than the RP specified by VT and in the range 90°-180° they were lower than the required value. Close to 90°, the VT-human performance qualitatively changed from overshooting to undershooting the RP specified by the VT. When 90° was specified by the VT, a local increase in variability occurred early in the Memory period—perhaps signaling a qualitative change in VT-human performance close to that particular value.

Because of its interesting features, we choose the 90° RP as the to-be-learned value for the next experiment. For ideal sinusoid, to achieve 90° RP the learner has to produce a peak in flexion that lags the flexion of the VT finger by one quarter of a cycle. Theoretically, the 90° RP is special because it constitutes a repellor, or instability point, in the coordination dynamics characterized with two 0° and 180° stable states. Yet, learning is expected to be facilitated at such instability points [50]. At Memory, the effect of Exposition did not last for long: when the coupling term C was set to zero, the learner rapidly shifted toward the spontaneously stable 0° RP. The memory of the just-performed pattern failed to overcome the spontaneous tendency of the VT-human system to produce 0° RP. In order to favor the persistence of learning the just-performed pattern we set both the HKB and modified Schöner-Kelso terms to zero. This step removes any attraction of the VT toward 0° or 180° RP.

Analysis of TE revealed that when the adjustment of the VT to the human learner changed, just after setting C at a zero value, the amount of information flowing from human to VT and from VT to human increased. This rise of TE coincided with the increase of RP variability. In the framework of a Shannonian information-theoretic approach, transfer entropy quantifies how much predictability about the next state of a target-partner is gained by knowing the past of a source-partner, *in addition* to what is already known about the target past [46]. This implies that if the target-partner’s next state is fully predictable from its own past, or from the current state of the source-partner, TE is about nil. This seems to have been the case at Exposition, where RP was stable. At the first instants of the Memory period, stability decreased. VT and learner positions were less predictable corresponding to a TE increase.

## Experiment 2

### Method

#### Participants

Eight unpaid volunteers, 6 males and 2 females, aged between 20 and 44 years took part in the study. None of them were involved in Experiment 1. They were self-proclaimed right-handers, naive to the purpose of the study, reported normal or corrected-to normal visual acuity and had no physical impairment impeding the production of required movement patterns. Participants provided written informed consent to research. The study was approved by the Internal Review Board at Florida Atlantic University and conformed to the principles expressed in the Declaration of Helsinki.

#### Methods and procedures

The apparatus, VT parameters and procedure were identical to Experiment 1with the following exceptions: For the entire experimental session, the RP specified by VT was set at Ψ = 90° and the required movement frequency at 1 Hz. During Exposition the parameter C was lowered to -3 in order to avoid too ‘passive’ a learning situation reputed for being detrimental to learning [51–53].The HKB coupling term was set to 0 (A = 0 and B = 0). Auditory feedback, a 410 Hz tone of 0.2s duration was introduced each time the target pattern of 90° (+/- 40°) was achieved by the human learner. The range of 40° was selected due to the weak discriminability of RPs close to 90° [32, 54]. The program allowed only one ‘reward’ or instance of auditory feedback to be delivered per movement cycle. In a 30 s trial, typically containing movements produced at a 1 Hz frequency, up to 20 auditory tones could be administered at Exposition and up to 10 tones at Memory. The experimental procedure was composed of five practice blocks, each containing ten trials. Blocks lasted about 9 min and were separated by a 2 min pause. The whole experimental session took about 60 min.

At the beginning of the experimental session, the procedure was explained to the participants. In addition to the instructions presented in Experiment 1, the role of feedback was explained. The task was presented like a game. Participants were instructed that when they heard the auditory tone, they would receive a point from their (virtual) partner signaling its satisfaction as the goal was achieved. They were told that only one reward would be delivered every cycle, and asked to obtain as many auditory tones as possible. The experimenter explained that the game was not a competitive but a collaborative game, in which the VT would help participants receive rewards. Participants should “just make their partner happy”. During practice trials, if participants were unable to receive any rewards, three instructions were repeated: “just keep trying”, “do not think too much, just enter in the game”, and “it is a collaborative game”.

#### Data reduction and analysis

Data reduction was identical to Experiment 1 except that the absolute value of constant error was used to capture whether the produced RP converged toward the required value. In addition, the number of hits of 90° RP at Exposition and Memory was computed. To simplify the comparison between Exposition and Memory, the number of hits actually achieved was expressed in percent of all possible hits. A 2 (Period = {Exposition, Memory}) × 5 (Block = {1, …, 5}) ANOVA on the percent of hits, RP, CE and circular variance was carried out with repeated measures on all factors. A similar 2 (Period) × 2 (Direction = {from VT to Human, from Human to VT}) × 5 (Block) ANOVA was carried out on TE. If necessary, this analysis was complemented with polynomial contrasts testing for the data trends.

### Results

#### Visual inspection of the data

**Fig 6:**
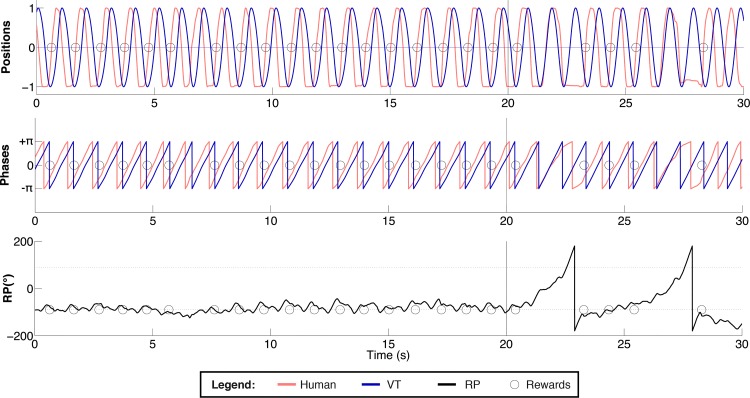
Individual practice trial. Normalized positions (top panel), phases (middle panel) and RP (bottom panel) are displayed as a function of time. Red lines pertain to human, blue to VT; the gray line depicts the RP specified by the VT (90° or -90°); the circles indicate the moment of feedback delivery. The vertical bar located at 20 s denotes the end of the Exposition and the beginning of the Memory periods.

Fig 6 displays an example of VT and human movement (top panel), corresponding phases (middle panel) and RP (bottom panel) in an early practice trial (11^th^). Circles denote the occurrences of 90° RP hits corresponding to the peak velocity of finger movement. At Exposition, the learner is able to produce the target pattern most of the time; at Memory, RP shifts, an experimental hallmark of weakened coupling. Nevertheless, the learner was capable of briefly returning to the RP specified by the VT thereby receiving some feedback, suggesting that the VT learning paradigm was working reasonably well.

**Fig 7:**
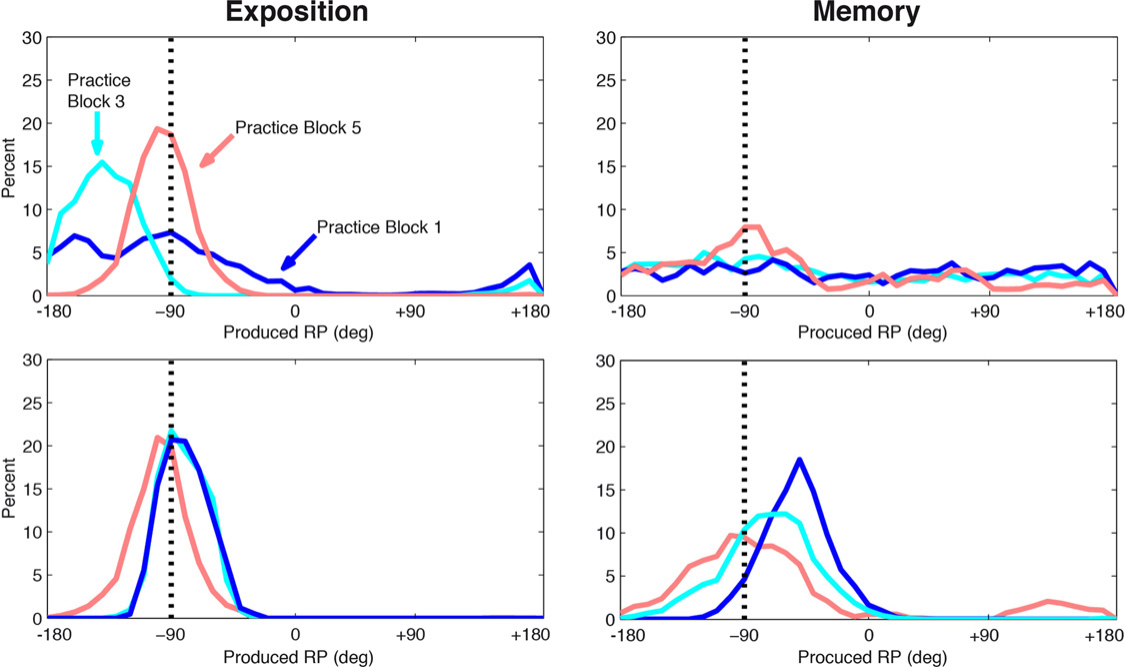
Learning a new Relative Phase. Individual distributions of produced RP during the first (blue line), third (cyan line) and last (red line) practice block for two individuals (top, and bottom panels, respectively). Left panels pertain to Exposition, right panels to Memory; the vertical dotted line represents the 90° RP specified by the VT. See text for details.

Fig 7 illustrates two individual learning paths. A learner (top panel) produced few RP specified by VT at the beginning of learning: the distribution of the produced RP is fairly flat for the first practice block. As practice proceeded, the produced RP converged around the RP specified by the VT. At Memory, RPs started to accumulate around the RP specified by the VT, but still with a large dispersion, even for the last practice block. Another learner (bottom panel) succeeded almost immediately to produce the required RP at Exposition and improved with practice: from the first practice block, the produced RP gathered around the RP specified by the VT, and accumulated there also at Memory.

#### Percent of hits

**Fig 8:**
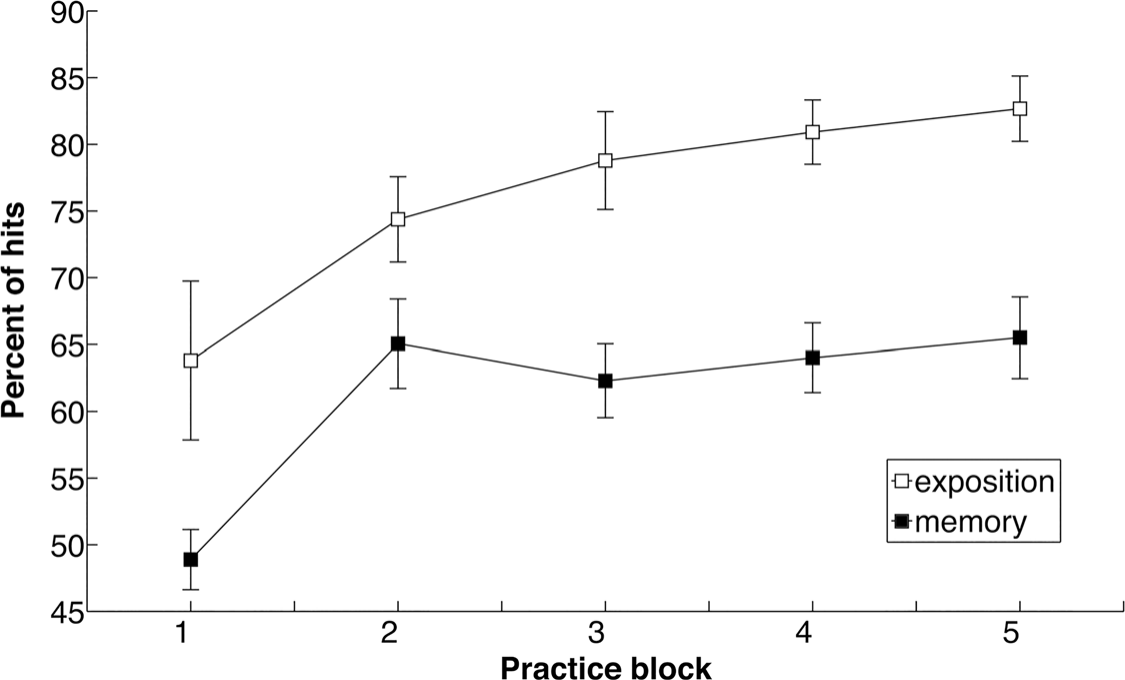
Reward-based performance. Percent of hits as a function of practice block at Exposition (open boxes) and at Memory (filled boxes).

The percent of hits as a function of practice is displayed in Fig 8. ANOVA revealed main effects of Period, *F*(1,7) = 7.87, *p <* 0.03,η^2^ = 0.53, and of Block, *F*(4,28) = 9.42, *p* < 0.01, η^2^ = 0.58. The percent of hits was higher in Exposition than in Memory. Polynomial contrasts revealed that percent hits increased linearly as a function of Block, *F*(1,7) = 12.35, *p* < 0.01, η^2^ = 0.638, both during Exposition and Memory periods.

#### Produced RP and absolute constant error

ANOVA on the produced RP revealed a main effect of Period only, *F*(1,7) = 22.09, *p* < 0.01, η^2^ = 0.53. The mean produced RP was -80.55° (SD = 7.86) at Exposition and -31.56° (SD = 11.49) at Memory. ANOVA on absolute constant error revealed a main effect of Period, *F*(F1,7) = 77.90, *p* < 0.01, η^2^ = 0.73, and of Block, *F* (4,28) = 4.97, *p* < 0.01, η^2^ = 0.53. Polynomial contrasts reported that constant error decreased linearly as a function of Block, *F*(1,7) = 7.22, *p* < 0.03, η^2^ = 0.44, dropping from 30.11° (SD = 30.12) to 18.31° (SD = 7.03) at Exposition, and from 54.51 (SD = 12.59) to 42.21 (SD = 12.81) at Memory.

#### Circular variance

ANOVA revealed a main effect of Period only, *F*(1,7) = 11.06, p < 0.01, η^2^ = 0.61. Circular variance was higher at Memory than in Exposition. Large inter-individual differences were present in the evolution of circular variance across trial blocks in Memory. Focusing on the first and last trial only, an additional A 2 (Block) × 2 (Period) ANOVA revealed a main effect of Period, *F*(1,7) = 13.71, *p* < 0.01, η^2^ = 0.66, and of Block, *F*(1,7) = 6.02, *p* < 0.04, η^2^ = 0.46. Circular variance decreased between the first and the last practice blocks.

#### Transfer entropy

**Fig 9:**
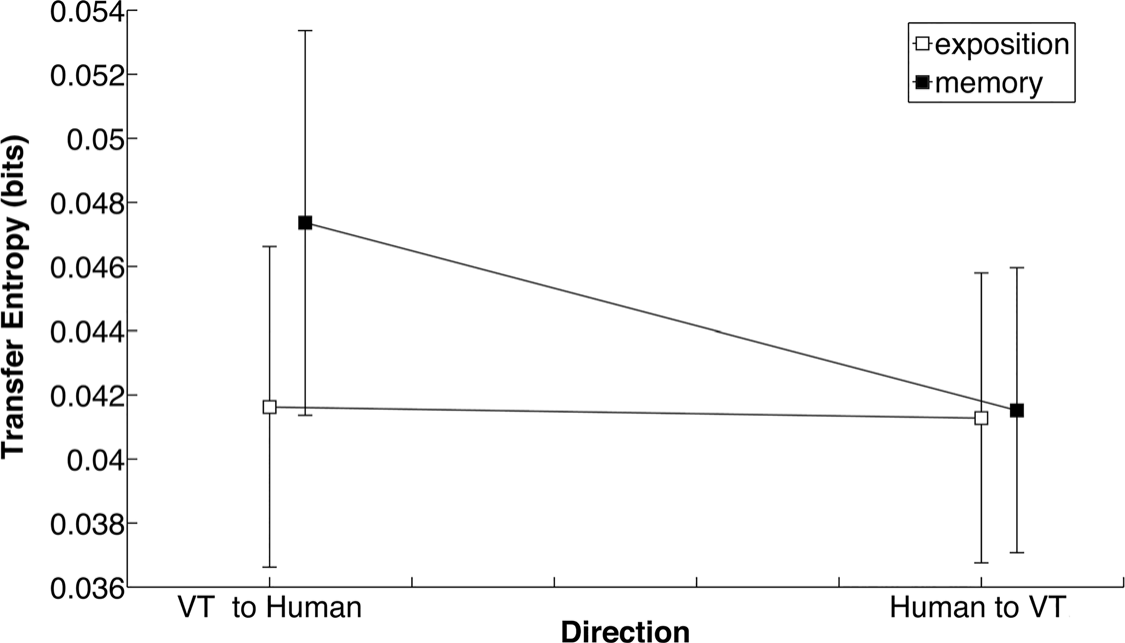
Information exchange during practice. Transfer entropy from VT to human and from human to VT at Exposition (open boxes) and at Memory (filled boxes).

Transfer entropy is displayed as a function of Direction ({from VT to Human, from Human to VT}) in Fig 9. ANOVA on TE revealed a main effect of Direction, *F*(1,7) = 7.36, *p* < 0.03, η^2^ = 0.51, and a Period × Direction interaction, *F*(1,7) = 8.15, *p* < 0.02, η^2^ = 0.54. Further t-tests showed that at Memory, TE was higher from VT to human than from Human to VT (*p < 0*.01).

### Conclusion and discussion

The percent of hits received by the VT-human system increased and absolute constant error decreased with practice, whereas the co-produced RP variability decreased between the first and the last practice block. Produced RP was closer to the value specified by VT when the teacher influenced the co-produced pattern (C≠0) compared to when the pattern was performed by the learner on his or her own (A = B = C = 0). These findings indicate: a) at Exposition the learner was able to adjust his/her performance to the (virtual) teacher in order to co-produce the required RP; and b) at Memory, the learner was able to persist in the just-produced RP on his/her own.

Again, transfer entropy from VT to human depended on RP variability, being lower at Exposition and higher at Memory. Note that when the HKB and the modified Schöner-Kelso coupling terms were cancelled (A = B = C = 0), there was no rise in the informational flow coming from the human to the VT. At first sight, it might be surprising that transfer entropy rises when the VT coupling, flowing from the human to the VT, is removed. Note, however, that transfer entropy refers only to the information coming from the past states of VT and not to the information flowing from the VT current state. Our results suggest that at Exposition, the learner mostly relies on the information coming from the VT current state, and at Memory on the information coming from the VT’s past.

## General Discussion

The aim of this paper was to establish that a virtual teacher (VT)–designed around specific models of coordination dynamics–can be used to enable humans to co-produce and learn new patterns of interpersonal coordination. Using a scanning procedure (Experiment 1), we showed that the VT can attract co-produced patterns toward any required relative phase, despite learners’ spontaneous tendencies to perform 0° and 180° RP. In Experiment 2, we demonstrated that a VT can help humans produce and memorize novel coordination patterns. Our results suggest that a mathematical model can be used as a conceptual and technical tool to teach human beings patterns of behavior that they might never have explored and learned on their own. As such, it puts into a new light the relationship between reality, models, and experimentation. Models are useful simplifications of reality: by excluding some processes they promote a deeper understanding of certain essential aspects. By combining a true reciprocal interaction and a rigorous control of half of the dyad, the Human Dynamic Clamp reveals new experimental effects, out-of-reach with traditional approaches using either model simulations or experimental observations [17–31].

By manipulating how the VT is coupled to a human partner, we showed that the amount of information coming from the partner’s past rises when the stability of the co-produced pattern drops. This drop appears when competition exists between the required RP and the spontaneous tendency to perform 0° displayed by the VT-human system (Experiment 1) or by the learner alone (Experiment 2). Resulting variability increases uncertainty which in turn creates room for uncertainty, or entropy, decrease and enhanced information transfer. Our findings provide further support for studies showing that variability (viz. low stability) enhances the transmission of information [55], see also [56]. Simulations with networks of coupled oscillators revealed that if network synchrony (viz. pattern stability) decreases, transfer entropy increases [57]. Such rises in variability have also been used to identify self-organizing pattern formation processes in the brain (e.g. see contributions in [58]).

Inter-personal coordination is a self-organizing process, in which information exchange binds participants into a macroscopic functional unit, developed under the distributed control of the partners. On the collective level, the coordination pattern is thought to exert a “top-down” constraint on the participants’ movements, inducing each of them to compensate for the momentary error of the other [59]. At the component level, individual participants tend to produce their spontaneously stable patterns, which may constitute a potential “bottom-up” threat to the stability of coordination. This threat increases as a function of the distance between the patterns intended by participants (cf. Experiment 1): when it is large, partners start struggling for coordination.

A difficulty we had to confront in our learning experiment was to select the value for parameters governing the strength of VT-human coupling. When partners share the same intention regarding the to-be-produced pattern, a strong bidirectional coupling enhances mutual compensation of errors on a short (millisecond) timescale [60]. In cases in which the intended patterns diverge, error compensation from the perspective of one participant produces error increases from the perspective of the other. During the learning trials, the learner must distinguish his/her own intended pattern from that intended by VT, and destabilize the former to stabilize the latter. Studies on human conversation reported that humans are prone to adopt a partner’s perspective when the co-actor is believed to be weakly adjustable [61]. This suggests that intermittent removal of all the coupling coming from VT (viz. Memory in Experiment 2) may be beneficial for learning.

A related issue pertains to the C value, the parameter determining how much the VT needs to adjust to the motion of the human in order to stabilize the RP specified in the model. On the one hand, too small a value of C allows the learner to dominate coordination and thus confine the produced RP close to the 0° or 180° patterns. On the other hand, too large a C value lets the VT dominate coordination too strongly, placing too much dependency on the teacher’s assistance–to the detriment of learning [52–54].

How much assistance a teacher should provide to learners is one of the oldest and hottest debates in the field of learning [62]. Our findings show that most of the information transfer takes place at the beginning of Memory, corresponding to the abrupt withdrawal of VT assistance. Further research is required to test how changes in coupling—gradual decreases or abrupt switches in the C parameter—promote information transfer and learning. The VT paradigm may facilitate our understanding of how teacher and learner tacitly and jointly modulate their relationship to best scaffold the learning process. An interesting possibility worth pursuing is that inter-personal learning is a nonlinear process in which spontaneous switching between two qualitatively distinct states involves teacher assistance and withdrawal, leading to trained or autonomous work on the part of the learner.

### Competition model of coordination learning

The present VT paradigm extends work on the learning of interlimb coordination [18–20, 33, 63] to the learning of interpersonal patterns. The VT paradigm makes use of the principles governing change of coordination in a single individual in order to gain better understanding of inter-personal coordination learning in social situations. Indeed, it has been shown that sensori-motor, inter-limb and interpersonal coordination follow the same general rules of coordination dynamics [3–13].

The process of learning via a VT follows the competition model [33] which formalizes individual learning as a shift process: when a person practices an initially unstable pattern while watching a visually displayed RP (Ψ), the to-be-learned RP enters in competition with the spontaneously stable RPs (0° and 180°). As a result, the learner modifies the latter by strengthening the shift process as practice proceeds. The shift model for new pattern learning was developed at the level of the RP dynamics [17](see Eq 2):

$$\dot{\varphi }=a\;\text{sin}(\varphi )+2bsin\left(2\varphi \right)+C\;sin(\psi -\varphi )$$ (2)

where *a* and *b* refers to parameters, φ to RP, Ψ is the to-be-learned RP, and C is the strength of intentional coupling. Dumas et al. [15] adapted the Schöner-Kelso intentional forcing term at the level of the limb dynamics (see Eq 3):

$$C_{int}\;=C\left(\left(\dot{x}-\dot{y}\right)cos\psi +\omega ysin\psi \right)$$ (3)

as illustrated in Fig 2. When VT successfully shifts the co-produced RP toward the specified value,Ψ the learner may produce a RP that he/she would never have produced on his own. Thereby conditions for practice and internalization of the to-be-learned RP are created, resulting in competition removal.

### Traditional RP learning and RP learning with VT

In traditional experimental designs, where the to-be-learned pattern is practiced by single individuals, two RPs are present: the visually displayed RP and the initially inaccurate RP produced by the learner [18]. The former is independent of the latter and the error between them can be assessed, a process known to be instrumental for motor learning [64]. In contrast, during the practice of the RP specified by the VT, only the co-produced RP is overtly present. Given that the overt RP is co-defined by both the VT and the learner and can be inaccurate, the learner does not have a fixed, absolute reference model upon which to compare his/her production in order to assess error. In the VT paradigm, it makes no sense to say that the learner flexed his finger 300 ms too late: were the finger flexed faster, by virtue of the bidirectional coupling, the VT would have altered its motion too! Only the feedback, delivered when the teacher-learner system happens to perform an accurate RP, informs the learner on the RP intended by the VT.

The function of feedback is debatable in our study. Traditionally, feedback offers most information when error is largest. In our study, feedback is provided only when the error criterion is reached, and error is small. Feedback may thus be envisioned to play a different role, promoting learning based on reinforcement rather than on error. The former leads to repeat the just-performed behavior, the latter to correct it. Recent evidence suggests that reinforcement-based and error-based learning are mediated by distinct cerebral processes [65].

Learning assisted by the VT is akin to weak learning by discovery, in which partners tacitly and jointly discover how to manipulate their relationship in order for the learner to discover the pattern to-be-learned. In the present work, feedback provides an explicit indication about the teacher’s intention. Further studies should test whether such explicit information can be removed. Recent work in social psychology suggests that motion alone may implicitly provide perceptual cues informing partners of the state of their interaction [66]. Information is coined as implicit when it is conveyed and captured involuntarily, unconsciously and automatically. On the one hand, the VT should ultimately be able to provide such implicit perceptual cues informing the learner about its intentions through VT motion alone. On the other hand, VT should be able to capture perceptual information from the learner’s motion about the learner’s state and needs. Recent studies on implicit human-computer interfaces [67] offer a first exploration of this issue.

In ecological learning situations [68], the greatest difficulty lies in the initial generation of a nearly accurate pattern: one learns nothing if one fails at each attempt! For learning to occur, the teacher must scaffold the learner to discover the behavior the learner would never have produced on his own. By fully controlling VT behavior, our paradigm provides a tool that offers insight into how teacher-learner systems can successfully solve this problem. The present findings speak to the efficiency of a learning method that involves switching between situations where the VT is coupled to the human or not. Others methods should be tested, such as gradually decreasing the coupling strength or the free selection of the coupling strength by the learner at each trial. An advantage of the VT is that this kind of issue may be readily addressed.

Virtual reality techniques have started to be used to promote perceptuo-motor learning [69].The VT may be envisioned as an instance of a teaching machine of mixed reality [70] or as a behavioral version of a brain-computer interface [71], providing always available assistance to the human with a rapidity, accuracy and consistency unachievable by a human teacher. Given that the VT design is principled, that is, based on non-arbitrary, empirically verified models of coordination dynamics and realistic animation, it should be straightforward in the future to transfer methods from the laboratory to more naturalistic situations. This opens up a set of possibilities for applied studies. For example, the VT might be exploited for new temporal pattern learning in sports and music which require sophisticated inter-personal patterns and/or to enhance the stabilization of unstable behavioral patterns during rehabilitation in disabled persons. The VT paradigm opens up new avenues for rehabilitation, complementary to existing behavioral intervention and closed-loop neuro-modulation therapeutics [72].

In summary, the main goal of the present research was to introduce the paradigm of a virtual teacher (VT) into studies of interpersonal learning. The VT is a specific version of the Human Dynamic Clamp and is grounded on an empirically verified model of behavioral stability and change. The movement of the human learner enters into the VT, animating an avatar displayed on a video screen and the coupling between avatar and human is bidirectional. We manipulated VT parameters and showed how the coordination between (virtual) teacher and learner allows for the co-production and stabilization of spontaneously unstable coordination patterns in memory.

## Appendix

### Transfer entropy

Transfer entropy (TE) is an information-theoretic measure developed by Schreiber (2000) to capture the amount and direction of information flow exchanged between two systems, X and Y. TE was specifically designed to overcome the limits of mutual information. Mutual information merely quantifies the amount of uncertainty about a random variable (e.g., X) reduced by the observation of another variable (e.g., Y). Transfer entropy, in addition, distinguishes how much X influences Y and how much Y influences X, because it is asymmetric under the exchange of X and Y.

To predict a next value of Y, y(i+τ), TE uses *k* previous samples of variable Y, composing Y past states vector, and *m* previous samples of variable X, composing X past states vector. Most intuitively, the formula of TE from X to Y may be represented as a difference between two conditional entropies [49] (Eq 1):

$$T_{X\to Y}=H\left(Y_{i+\tau }|Y_{i}^{\left(k\right)}\right)-H\left(Y_{i+1}|Y_{i}^{\left(k\right)},X_{i}^{\left(m\right)}\right)$$ A1

Where i indicates a given instant, and τ the time lag. Initially, Schreiber (2000) had formulated TE as (Eq 2):

$$T_{X\to Y}=\sum_{x,y}\;p\left(y_{i+\tau },y_{i}^{\left(k\right)},x_{j}^{\left(m\right)}\right)log\frac{p\left(y_{i+\tau }|y_{i}^{k},x_{i}^{m}\right)}{p\left(y_{i+\tau }|y_{i}^{k},\right)}$$ A2

where the expression before the logarithm pertains to joint probability. For computation purposes, it is useful to rewrite TE as (Eq 3):

$$T_{X\to Y}=\sum_{x,y}\;p\left(y_{i+\tau },y_{i}^{\left(k\right)},x_{j}^{\left(m\right)}\right)log\frac{p\left(y_{i+\tau },y_{i}^{\left(k\right)},x_{j}^{\left(m\right)}\right)p\left(y_{i}^{\left(k\right)}\right)}{p\left(y_{i}^{\left(k\right)},x_{j}^{\left(m\right)}\right)p\left(y_{i+\tau },y_{i}^{\left(k\right)}\right)}$$ A3

### EQUATIONS

$$T_{X\to Y}=H\left(Y_{i+\tau }|Y_{i}^{\left(k\right)}\right)-H\left(Y_{i+1}|Y_{i}^{\left(k\right)},X_{i}^{\left(m\right)}\right)$$ Equation 1

$$\dot{\varphi }=a\;\text{sin}(\varphi )+2bsin\left(2\varphi \right)+C\;sin(\psi -\varphi )$$ Equation 2

$$C_{int}=C\left(\left(\dot{x}-\dot{y}\right)cos\psi +\omega ysin\psi \right)$$ Equation 3
